# The Improvement of Porcine In Vitro Embryo Development through Regulating Autophagy by miRNA-143 Inhibition

**DOI:** 10.3390/ani12192651

**Published:** 2022-10-02

**Authors:** Muhammad Rosyid Ridlo, Eui Hyun Kim, Eun Pyo Kim, Geon A. Kim

**Affiliations:** 1 Department of Theriogenology and Biotechnology, Research Institute for Veterinary Science, College of Veterinary Medicine, Seoul National University, Seoul 08826, Korea; 2 Department of Bioresources Technology and Veterinary, Vocational College, Universitas Gadjah Mada, Yogyakarta 5281, Indonesia; 3 Department of Biomedical Laboratory Science, College of Health Science, Eulji University, Uijeongbu 11759, Korea

**Keywords:** autophagy, ER-phagy, ER stress, embryo, miR-143, porcine

## Abstract

**Simple Summary:**

The improvement of in vitro embryo development is an important factor for the advancement of reproductive technology. Recently, studying microRNA related to early embryos is a strategic approach. This research applies miR-143 (mimics and inhibitors) to the porcine parthenogenetically activated embryos. Interesting results are revealed in this study: the miR-143 inhibitor improves the embryo development to the blastocyst. In addition, our research reveals the enhancement of autophagy and ER-phagy associated markers with higher levels of gene expression.

**Abstract:**

In vitro embryo research is an important stage for the advancement of many reproductive technologies in research and agriculture. For this reason, the improvement of in vitro embryo development is a strategic field worthy of investigation. Relatively little is known about miR-143 and its effects on autophagy associated with embryo development and in vitro embryo culture. In this study, we examined the effect of miR-143 (via mimics and inhibitors) on embryonic development threatened by microinjection after parthenogenetic activation. We evaluated rates of cleavage, blastocyst, and total cell number of blastocyst; additionally, we performed LC3 immunofluorescence analysis and mRNA expression analyses of genes associated with autophagy, endoplasmic reticulum (ER)-phagy, ER stress, embryo quality, and apoptosis. The inhibition of miR-143 positively influenced embryo development by increasing the activity of autophagy and ER-phagy and the expression of embryo quality-related genes, while reducing apoptosis. In contrast, treatment with miR-143 mimics increased ER stress-related gene expression and apoptosis, and reduced embryo development. Together, our findings indicate that miR-143 plays a role in the interplay between autophagy, ER-phagy, and embryo quality during early porcine embryo development.

## 1. Introduction

Recently, microRNAs (miRNAs) have been revealed to perform strategic functions in cellular function, gene expression, reproduction, and clinical disease [1,2]. miRNAs are short noncoding RNAs, approximately 17–25 nucleotides long [2,3], that influence the expression of specific genes by suppressing mRNA translation, thereby decreasing or indirectly increasing the levels of numerous mRNAs [3,4,5]. In reproduction, the availability of miRNAs during early embryo stages has been investigated as a factor connecting interactions between the mother, oogenesis, spermatogenesis, embryogenesis, and folliculogenesis [5,6]. The existence of miRNAs has been reported in several stages of embryo development. miR-143, -320, and -101 were identified in conditioned media from murine preimplantation embryos [7]. During porcine oogenesis, miRNAs such as miR-27b-3p, miR-143, miR-183, miR-10b, and miR-486 were detected in geminal vesicles and metaphase II oocytes [8]. In addition, miR-125 and miR-128 were detected in early stages of embryogenesis and miR-129, -92, -210, -1246, -378d, and miR-21 were found in porcine blastocysts [7,8,9,10]. 

A recent study in patients with ovarian disease noted that miR-143 was highly expressed in the follicular fluid and further increased apoptosis by inhibiting the Smad1/5/8 pathways [11]. Research on gastrointestinal disease showed that miR-143 exerted a negative effect, triggering inflammation through the regulation of autophagy by targeting ATG2B [12]. These findings imply that miR-143 is associated with both inflammation and autophagy. In addition, miR-143 was shown to downregulate autophagy, but upregulate proinflammatory cytokines such as IL-8 and IFN-γ [12,13]. Endoplasmic reticulum (ER)-phagy is a part of the autophagy process that is important for balancing the cellular environment. 

However, comparatively little is known about the role of miR-143 in autophagy associated with in vitro embryo culture and embryonic development. The aim of our research was therefore to investigate the effect of miR-143 (mimic and inhibition) on embryonic development threatened by microinjection after parthenogenetic activation. Our findings revealed that the inhibition of miR-143 increased blastocyst cells numbers, and upregulated autophagy and ER-phagy activity, as well as increased the expression of embryo quality related genes, while reducing apoptosis. In contrast, treatment with miR-143 mimicked increased ER stress-related gene expression and apoptosis, and reduced embryo development.

## 2. Materials and Methods

The chemicals used for this experiment were applied using products from the Sigma-Aldrich chemical company (St. Louis, MO, USA).

### 2.1. Collecting and Preparing the Oocytes

Collection of the prepubertal gilt ovaries were performed in the local abattoir and moved to the laboratory utilizing saline at 31–37 °C. The follicles of ovaries, 4–6 mm in diameter, were aspirated by a 10 mL syringe and 18 G needle. The cumulus oocytes complexes (COCs) were placed in a petri dish and rinsed three times by using medium consisting of 9.5 g/L of TCM-199 powder (Cat. 31100-027, Thermo fisher scientific, Waltham, MA, USA), 1% penicillin-streptomycin (Invitrogen), 0.3% PVA, 2 mM natrium bicarbonate, 5 mM natrium hydroxide, and 10 mM HEPES. Further, the COCs with at least three layers of cumulus cells and darkened homogeneous cytoplasms were selected for IVM [14,15]. The total duration of IVM for the oocytes was 44 h in 39 °C, 95% air humidity, and 5% CO_2_. The IVM medium containing numerous ingredients, including TCM-199 liquid form (Cat. 11150.059), 0.57 mM cysteine, 10 ng/mL epidermal growth factor, human chorionic gonadotropin and equine chorionic gonadotropin at 10 IU/mL each, 10% porcine follicular fluid, 0.91 mM sodium pyruvate, and 10 μL/mL insulin-transferin-selenium mixture solution. In addition, after 22 h of maturation, the IVM medium was changed for hormone-free maturation medium until 44 h. 

### 2.2. Outline of the Experiment

The purpose of this investigation was to study the effectivity of microinjection on miR-143 treatment, including inhibition and mimicry of miR-143. The experiment was designed in four group treatments as follows: (a) microinjection of zygote with diethylpyrocarbonate water (DEPC water) as a control, (b) microinjection of scramble RNA, (c) microinjection of miR-143-inhibitor, and (d) microinjection of miR-143-mimic. Numerous parameters were evaluated in this study: The first evaluation was the assessment of embryo development, including cleavage and blastocyst formation percentage, and total cell number of the blastocyst. The second evaluation was the assessment of LC3 protein expression in cleavage stage at Day 2 and of the blastocyst at Day 7. Gene expression related to ER-phagy, ER stress, embryo quality, and apoptosis markers were analyzed in the third evaluation. 

### 2.3. Parthenogenetic Activation of Porcine Oocytes

Immediately after IVM of the porcine oocytes, the COCs were denuded by 1% hyaluronidase using a pipette. Afterwards, the selected oocytes were moved to activation medium containing mannitol 0.28 M, HEPES 0.5 mM, MgSO_4_ 0.1 mM, and CaCl_2_ 0.1 mM. Then, the oocytes were placed in a chamber connected to the electric parthenogenetic activation machine BTX-2001 (BTX Inc., San Diego, CA, USA). Next, the oocytes were cultured in porcine zygote medium-5 (Wako Chemicals, Osaka, Japan, Cat. CSR-CK024). The incubator setting was 39 °C, 90% N_2,_ 5% O_2,_ and 5% CO_2_.

### 2.4. Microinjection

Microinjections of the zygotes were performed 6 h after the electric activation of the oocytes. The zygotes were moved to a 4 μL drop of PZM-5 covered with mineral oil for the microinjection process which was explained previously [16,17]. In brief, the zygotes were injected using a microscope (Eclipse TE2000-S, Nikon, Tokyo, Japan) connected to the microinjection machine Femtojet (Eppendorf, Hamburg, Germany). According to the previous study, the injection of microRNA with concentration 20 pmol/μL was proven by observation of cytoplasmic movement due to injection [16,18]. The microRNA chemicals applied were miR-143 mimic, inhibitor, and scramble designed by Bioneer (Daejon, Korea). The information related to the micro-RNA is presented in Table 1. Further, the zygotes were returned for the continuation of IVC until Day 7. 

### 2.5. Assessment of Embryo Development

The evaluation of embryo development was performed on Day 2 (48 h) for cleavage rate and on Day 7 (168 h) for blastocyst formation rate and total cell number (TCN); the day of oocyte activation was counted as Day 0. The TCN of blastocyst evaluation was performed by utilizing 5 μg/mL bisbenzimide or Hoechst-33342 for 10 min in a dark location. Then the blastocysts were placed in a drop of glycerol and covered by a cover glass for evaluation under a fluorescence microscope (Nikon Corp, Tokyo, Japan). Image analysis was implemented using Image J software (National Institute of Health, Bethesda, MD, USA).

### 2.6. Immunofluorescence Staining of Embryo

In this experiment, we utilized the immunofluorescence technique by using LC3 as a primary antibody. The protocol for antibody staining was described previously [18,19,20]. In brief, the embryos were placed in 4% paraformaldehyde diluted in PBS at room temperature for 1 h. Then, the embryos were incubated in 1% Triton X-100 diluted in distilled water at s39 °C for at least 1 h. Next, the samples were moved into the LC3 primary antibody diluted with 2% BSA overnight in 4 °C (1:350; PA1-46286, Invitrogen, IL, USA). Immediately after incubation in the primary antibody solution, the samples were placed into secondary antibody goat anti-rabbit IgG H&L (Texas Red ^®^, 1:250, ab6719; Abcam, Cambridge, UK) in 2% BSA diluted with PBS in a dark location at 25 °C for 2 h. Further, the final process was staining the nuclear staining with Hoechst-33342 for 10 min in the dark location at 25 °C. 

### 2.7. Gene Expression Analysis via qRT-PCR

The implementation protocol of qRT-PCR has been previously explained [20,21,22]. The samples were obtained from at least 400 embryos in the cleavage stage (Day 2), nine biological replicates for each group. Then, the samples were processed for RNA extraction, complementary DNA (cDNA) synthesis, and mRNA expression analysis via a StepOne™ qRT-PCR machine (Applied Biosystems, Singapore). The mixture of the reaction solution for qRT-PCR was set as follows: 0.4 μL for each primer of forward and reverse, 10 μL SYBR green Premix Ex Taq (Takara, Otsu, Japan), 1 μL of cDNA sample, and 8.2 μL nuclease-free water. The mixture reaction was gently pipetted into a 96-well plate for PCR (Micro-Amp Optical 96 well, Applied Biosystems, Singapore). The cycle was repeated 40 times, the denaturation sample was kept at 95 °C for 15 s, the annealing process lasted 1 min at 60 °C, and the extension period lasted 1 min at 72 °C. The implementation of PCR analysis was performed at least three times. The GAPH gene was utilized as an endogenous gene. The primer genes used in this experiment are presented in Table 2. In addition, the relative mRNA expression evaluation was calculated by using the formula R = 2 − ^[ΔCt sample − ΔCt control]^.

### 2.8. Data Analysis

Statistical analysis of the data experiments was evaluated by utilizing a GraphPad Prism 5. In this experiment, the data were evaluated via univariate of variance (ANOVA) and Tukey’s tests. In addition, any values at *p* < 0.05 were termed as statistically significant differences.

## 3. Results

### 3.1. Evaluation of Embryo Growth after miR-143 Microinjection 

We first examined the development of porcine embryos after microinjecting them with miR-143 inhibitor, mimic, or scrambled sequences, with DEPC as a control, and evaluated TCN, cleavage, and blastocyst percentages (Table 3). The results demonstrated that the miR-143 inhibitor group yielded a significantly higher percentage in cleavage, blastocyst, and TCN of blastocyst compared other groups (*p* < 0.05). Further analysis revealed that the results from the control and scrambled groups were comparable, while treatment with an miR-143 mimic yielded significantly lower embryo and blastocyst numbers compared to the other groups (*p* < 0.05).

### 3.2. Effect of miR-143 Microinjection on Autophagy in Porcine Embryos

**Figure 1:**
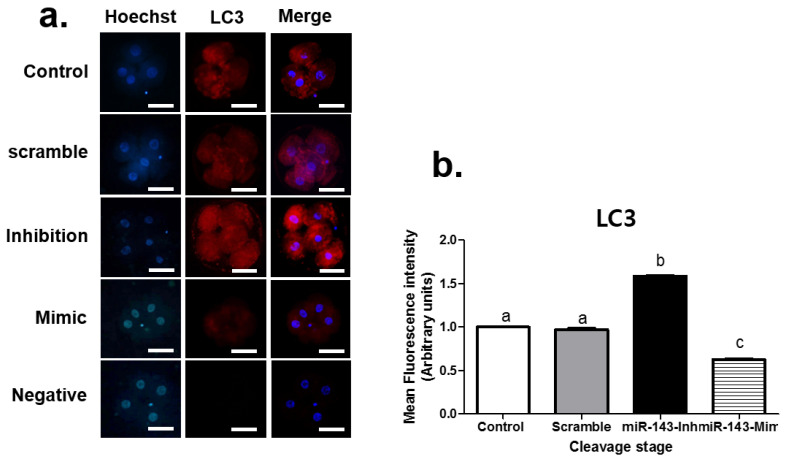
Expression of LC3 immunostaining in cleavage stage embryos on Day 2 after microinjection with miR-143. (**a**) Representative fluorescence images of LC3 expression and Hoechst staining. In addition, merge is the combination figure of Hoechst and LC3 staining. (**b**) Graph showing quantitation of LC3 fluorescence intensity. Data are presented as standard error of the mean (±SEM), with each experiment performed using at least 24 embryos per group, obtained from four biological replicates. Groups labeled with different lowercase letters indicate statistically significant differences (*p* < 0.05). Scale bars = 50 μm, magnification = 400×. miR-143-Inh = microRNA 143-Inhibitor, miR-143-Mim = microRNA 143-Mimic.

**Figure 2:**
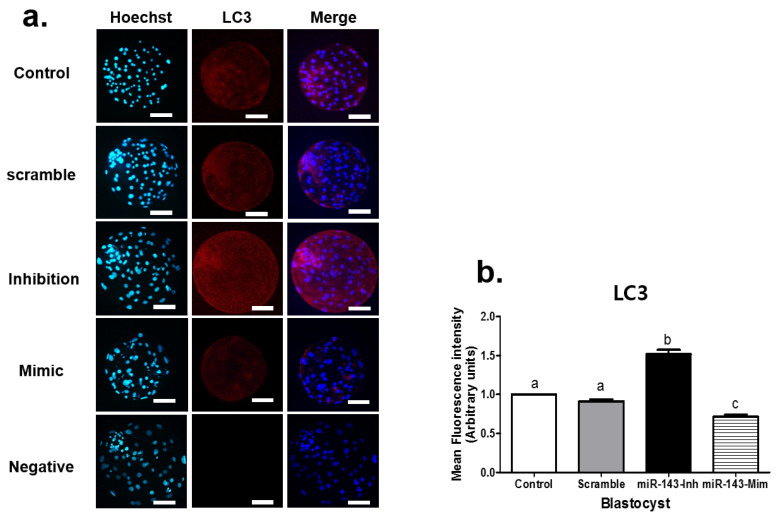
LC3 immunostaining in blastocyst stage embryos on Day 7 after microinjection with miR-143. (**a**) Representative fluorescence images of LC3 expression and Hoechst staining. In addition, merge is the combination figure of Hoechst and LC3 staining. (**b**) Graph showing quantitation of LC3 fluorescence intensity. Data are presented as standard error of the mean (±SEM), with each experiment performed using at least 24 embryos per group, obtained from four biological replicates. Groups labeled with different lowercase letters indicate statistically significant differences (*p* < 0.05). Scale bars = 50 μm, magnification = 400×. miR-143-Inh = microRNA 143-Inhibitor, miR-143-Mim = microRNA 143-Mimic.

We next investigated the effect of miR-143 microinjection on LC3 protein expression at the cleavage (Figure 1) and blastocyst (Figure 2) stages. The group injected with miR-143 inhibitors exhibited the highest LC3 fluorescence intensity among the groups, while conversely, miR-143 mimic treatment decreased the intensity of fluorescent LC3 staining (*p* < 0.05). As observed in the previous experiment, the results of treatment from the scrambled sequence were comparable to those of the control group (*p* > 0.05). 

### 3.3. Gene Expression Analysis of Embryos Microinjected with miR-143

**Figure 3:**
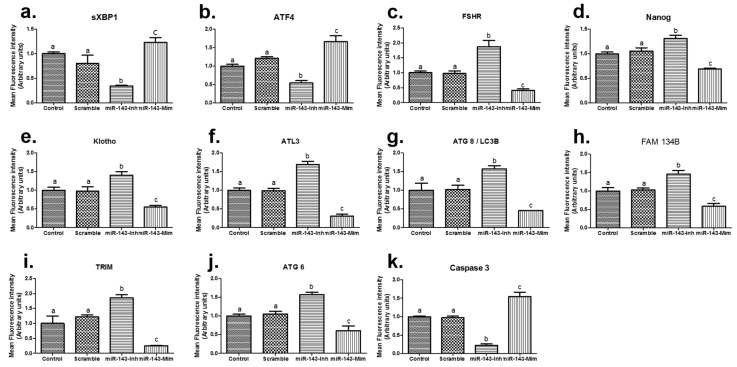
mRNA expression levels of genes associated with ER stress, ER-phagy, embryo quality, and apoptosis in cleavage stage embryos on Day 2 after microinjection (**a**–**k**). Data are presented as standard error of the mean (±SEM). The experiment was performed using at least three technical replicates during qRT-PCR analysis, on mRNA obtained from at least 400 embryos per sample and nine biological replicates. Groups labeled with different lowercase letters indicate statistically significant differences (*p* < 0.05). Scale bars = 50 μm, magnification = 400×. miR-143-Inh = microRNA 143-Inhibitor, miR-143-Mim = microRNA 143-Mimic.

We next investigated the mRNA expression levels of genes associated with ER stress (*sXBP1* and *ATF4*), embryo pluripotency and quality, apoptosis (*Caspase3*), and autophagy and ER-phagy (*ATL3, LC3B, FAM134, TRIM*, and *ATG6*) (Figure 3). The results demonstrated that treatment with the miR-143 inhibitor significantly reduced the expression of ER stress-related genes (*sXBP1* and *ATF4*), whereas the miR-143 mimic significantly upregulated *sXBP1* and *ATF4.* The miR-143 inhibitor also significantly increased the gene expressions of *Nanog*, *Klotho,* and *FSHR* (*p* < 0.05). The analysis of autophagy and ER-phagy-related genes revealed that miR-143 inhibitor treatment significantly upregulated these genes (*ATL3, LC3B, FAM134, TRIM*, and *ATG6*) compared to the control embryos. However, treatment with the miR-143 mimic significantly decreased the expression of *Nanog*, *Klotho*, and the autophagy- and ER-phagy-associated genes compared to the other groups. 

## 4. Discussion

Numerous studies have revealed that miRNAs have strategic functions at various cellular levels, including the development of early-stage embryos. In addition, miRNAs are involved in various organ systems, including the reproductive and circulatory systems in the body [5,22]. 

It has been shown that many epigenetic factors, including DNA methylation and histone modification, affect porcine early embryo development [23,24,25], suggesting that miRNAs may also regulate the development of the early embryo [23]. Liu et al. demonstrated that miR-34c is responsible for initiating the first cleavage divisions in murine embryos [26]. Furthermore, numerous miRNAs have been detected in zygotes and early embryos from pigs [10,27]. However, comprehensive analyses of the links and mechanisms governing the roles of miRNAs in early porcine embryos are still required. However, Ridlo et al. have previously shown that miR-210 inhibition reduces both apoptosis and the expression of ER stress-associated genes such as *XBP1, ATF4,* and *PTPN1* in early-stage porcine embryos. In contrast, treatment with the miR-210 mimic resulted in negative effects on early porcine embryos in vitro [18]. 

During in vitro embryo development, oxidative stress or ER stress are the main reasons for poor embryo development. Data from our previous study indicated that ER stress is involved in embryo development and is regulated by an miR210 inhibitor, which raised the possibility that suppression of ER stress by another microRNA may promote embryo development in vitro. Furthermore, autophagy is a stress adaption that eliminates damaged cellular components, and it plays an important role in determining the viability of cells under oxidative stress. Promoting autophagy may thus enhance the resistance of cells to oxidative stress [28]. In addition, various miRNAs play roles in the activation of autophagy. Various studies have noted that miR-9, miR-17, and miR-124 are closely involved in autophagy activity. The authors showed that the initiation of autophagy was triggered by the activation of AMPK, which was in turn activated by these miRNAs (miR-9, miR-124, and miR-17) [29,30,31]. 

A recent study of trophoblast cells demonstrated that increasing levels of miR-143 repressed metastasis and proliferation in cancer cells, and increased cell apoptosis [32]. miR-143 has also been associated with inflammation. Lin et al. reported that miR-143 has a strong involvement in inflammation and autophagy-associated downregulation of gene expression in inflammatory bowel disease. miR-143 suppressed ATG2 expression, a well-known mediator of autophagy [12]. 

Our experimental results indicated that miR-143 exerts significant effects on the development of embryos at the cleavage and blastocyst stages, and on total cell numbers of blastocysts. In this experiment, miR-143 inhibition enhanced the cleavage, blastocyst numbers, and TCN of blastocyst percentage when compared to all of the other groups. This implies that there were beneficial effects when an miR-143 inhibitor was injected into the porcine zygotes derived from parthenogenetic activation. Conversely, treatment with the miR-143 mimic revealed a negative influence on embryo development, with reduced cleavage, blastocyst numbers, and TCN of blastocyst percentage rates exhibited by embryos in this group compared to the control. 

Analysis of the autophagy-related protein LC3 via immunofluorescence staining showed that miR-143 inhibition significantly upregulated LC3 expression at the cleavage and blastocyst stages, while the miR-143 mimic reduced LC3 intensity at these stages. These findings imply that miR-143 is associated with autophagy in early-stage in vitro porcine embryos. The integration autophagy factors, like LC3 and ATG8, initiate the autophagy and further proses forming the autophagosome formation [33,34]. This implies that the LC3 is an indicator of autophagy activity. 

Molecular RT-PCR analysis revealed that miR-143 treatment of activated oocytes influenced mRNA expression in early-stage porcine embryos. miR-143 inhibition resulted in the downregulation of ER stress-associated genes such as *sXBP1* and *ATF4*. Interestingly, the mRNA expression levels of genes associated with autophagy (*ATG8/LC3, TRIM*, and *ATG6*) and ER-phagy (ATL3 and FAM134B) were upregulated in comparison to the control group when miR-143 was inhibited. These results confirmed that the inhibition of miR-143 enhanced autophagy and ER-phagy activity. Moreover, this treatment also increased the expression of genes related to aging capacity (*Klotho*) and embryo quality (*Nanog*), suggesting a link between autophagy, ER-phagy, embryo quality, and embryo development. 

Further analysis revealed that treatment of activated oocytes with the miR-143 mimic increased the mRNA expression level of ER stress-related genes such as *sXBP1* and *ATF4*, and decreased the expression of autophagy and ER-phagy-related genes (*ATL3, ATG8, FAM134, TRIM* and *ATG6*), as well as *Klotho* and *Nanog*. Treatment with the miR-143 mimic revealed negative effects on cleavage, blastocyst number, and TCN of blastocyst percentage, as well as revealing increased activity of ER stress-related genes. Concurrently, the miR-143 mimic decreased autophagy- and embryo quality-related genes. 

When we analyzed the effects on apoptosis, we observed that miR-143 inhibition resulted in a reduction of the apoptosis-related gene, *Caspase3,* while the miR-143 mimic increased expression of *Caspase3*. This finding is supported by those from a previous study demonstrating that miR-143 triggered apoptosis by inhibiting the Smad1/5/8 pathways and regulating the expression of BMP-specific type 1 (*BMPR1A*) [11]. In addition, an examination of *FSHR*, a gene target of miR-143 [6], revealed that miR-143 inhibitors and mimics increased and decreased the mRNA expression of *FSHR*, respectively. 

Our data also revealed interesting results regarding the expression of ER stress-associated genes. miR-143 mimics increased *sXBP1* and *ATF4* expression, while miR-143 inhibition downregulated the expression of these genes and improved the embryo development. These results indicate that miR-143 inhibition reduces ER stress. In contrast, the miR-143 mimic has a negative effect on embryo development and aggravates ER stress in these embryos. However, deeper investigation related to expression levels of miR-143 in the early development of porcine embryos, especially in the parthenogenetic embryos, is important and necessary in future study. 

## 5. Conclusions

Our findings revealed that the inhibition of miR-143 exerted a positive effect on embryogenesis by increasing the activity of autophagy and ER-phagy, and the expression of genes associated with embryo quality, as well as improving embryo development and reducing apoptosis. Furthermore, treatment with an miR-143 mimic increased ER stress and apoptosis, and reduced embryo development. Our investigation demonstrates that miR-143 plays important roles in early porcine embryo development in vitro.

## Figures and Tables

**Table 1:** micro-RNA-143 and scramble sequences.

Product Number	Micro-RNA	Sequence (5′-3′)	Tm	Base Count
rna-double-Customorder	miR-143 mimic	UGAGAUGAAGCACUGUAGCUC	73.8	21
		GAGCUACAGUGCUUCAUCUCA		
rna-single-Customorder	miR-143 inhibitor	GAGCUACAGUGCUUCAUCUCA	73.8	21
rna-double-Customorder	Scramble	CGAACAGAUAAAGCCGCUGUAAGUA	-	25
		UACUUACAGCGGCUUUAUCUGUUCG		

**Table 2:** Primer gene sequences information for mRNA expression analysis.

Genes	Primer Sequences	Accession Number	Product Size (bp)
*sXBP1*	F: GGAGTTAAGACAGCGCTTGG R: GAGATGTTCTGGAGGGGTGA	NM_001271738.1	142
*ATF4*	F: AGTCCTTTTCTGCGAGTGGG R: CTGCTGCCTCTAATACGCCA	NM_001123078.1	80
*FSHR*	F: TTCACAGTCGCCCTCTTTCC R: ACGTACAGCTGTGACAAGGG	NM_214386.3	101
*Nanog*	F: GGTTTATGGGCCTGAAGAAA R: GATCCATGGAGGAAGGAAGA	NM_01119971	98
*Klotho*	F: GCTACAGC ATCAGA CGTGGA R: TCCCTT CTAGGGG CTGATTT	XM_021065566.1	147
*ATL3*	F: TCGAGGAAGTACAGGTCGGT R: TGGCATCCCTACCACTCT GA	XM_021082904.1	127
*ATG8/LC3*	F: GGGCGTAGGAGACACAAGAG R: AAGGTTTTCTCGGACGGCAT	NM_001190290.1	134
*FAM134B*	F: TTGCCCACTGAGCTCAAGAG R: CACTGCTCAGAGGAAGGGTG	XM_003483804.4	103
*TRIM*	F: AAAACCCGATTGCTTTGCCC R: CTTCCAGCAGCTCCATCACA	XM_021065576.1	91
*ATG6*	F: AGGGAGCTGGCATTAGAGGA R: AGCCTGGACCTTCTCGAGAT	NM_001044530.1	99
*Caspase3*	F: GCCATGGTGAAGAAGGAAAA R: GGCAGGCCTGAATTATGAAA	NM_214131.1	132
*GAPDH*	F: GTCGGTTGTGGATCTGACCT R: TTGACGAAGTGGTCGTTGAG	NM_001206359	207

mRNA, messenger RNA; bp, base pair; F, forward; R, reverse.

**Table 3:** Evaluation of development in embryos microinjected with miR-143.

Group	Number of Embryos	Number of Embryos Developed (Mean ± SEM, %)		Total Blastocyst Cell Number (Mean ± SEM)
		≧2 Cells	Blastocyst	
Control	192	151 (78.65 ± 0.56) ^a^	38 (20.03 ± 0.9) ^a^	63.13 ± 0.65 ^a^
Scramble	189	147 (77.74 ± 0.74) ^a^	36 (19.15 ± 0.63) ^a^	61.33 ± 0.64 ^a^
miR-143-inhibition	191	167 (87.54 ± 0.35) ^b^	60 (31.58 ± 1.50) ^b^	76.21 ± 0.71 ^b^
miR-143-mimic	189	126 (66.31 ± 1.76) ^c^	24 (13.04 ± 1.43) ^c^	46.08 ± 0.44 ^c^

SEM: Standard error of the mean; miR-143: microRNA 143; number of replicates in embryo development experiment = 6. At least 36 embryos were utilized for total cell number (TCN) analysis using the Hoechst technique, each replicate = 6 embryos; differing lowercase letters (a, b, c) in the same column indicate a significant difference (*p* < 0.05).

## Data Availability

Not applicable.
